# Prévalence et facteurs associés à l’anémie en grossesse à l’Hôpital Général de Douala

**DOI:** 10.11604/pamj.2016.25.133.10610

**Published:** 2016-11-04

**Authors:** Charlotte Nguefack Tchente, Eveline Ngouadjeu Dongho Tsakeu, Arlette Géraldine Nguea, Théophile Nana Njamen, Gregory Halle Ekane, Eugene Belley Priso

**Affiliations:** 1 Faculté de Médecine et des Sciences Pharmaceutiques, Université de Douala, Douala,Cameroun; 2 Service de Gynécologie-Obstétrique, Hôpital Général de Douala, Douala,Cameroun; 3 Service d’Hématologie, Hôpital Général de Douala, Douala,Cameroun; 4 Faculté des Sciences de la Santé, Université de Buéa, Buéa, Cameroun; 5 Faculté de Médecine et des Sciences Biomédicales, Université de Yaoundé, Yaoundé, Cameroun

**Keywords:** Anémie, grossesse, prévalence, facteurs associés, Anemia, pregnancy, prevalence, associated factors

## Abstract

**Introduction:**

L’anémie est un problème de santé publique, prédominant chez les enfants et les femmes en âge de procréer. L’objectif de l’étude était de déterminer la prévalence et les facteurs associés à l’anémie chez les femmes enceintes à l’Hôpital Général de Douala.

**Méthodes:**

Il s’agissait d’une étude transversale qui s’est déroulée de juillet 2012 à juillet 2013. Toutes les femmes enceintes consentantes se présentant pour consultation prénatale et ayant réalisées une numération formule sanguine (NFS) étaient incluses. Les caractéristiques sociodémographiques, les antécédents obstétricaux et les résultats de la NFS étaient enregistrés sur une fiche technique pré-testée. L’anémie était définie selon les critères de l’OMS. Après quelques statistiques descriptives, nous avons effectué une analyse bivariée à l’aide du test de Chi 2 et la probabilité exacte de Fisher pour rechercher les facteurs associés à l’anémie. Une valeur de p< 0,05 était considérée significative.

**Résultats:**

Au total 415 gestantes ont été recrutées. La prévalence de l’anémie était de 39,8%. L’âge moyen était de 29,89±4,835ans. Le taux moyen d’hémoglobine était de 10,93±1,23.L’anémie normochrome normocytaire (53,3%) était prédominante. L’anémie était sévère dans 2,4% des cas. L’anémie en grossesse était significativement associée aux antécédents de pathologies chroniques (P=0,02) et d’anémie gravidique antérieure (P=0,003). L’anémie était plus observée au 3^ème^ trimestre (P=0,04) et l’allaitement maternel était protecteur (P=0,02).

**Conclusion:**

La prévalence de l’anémie chez la femme enceinte reste élevée. Un accent doit être mis sur une meilleureprise en charge des pathologies chroniques chez les gestantes et sur leur suivi en post natal afin de corriger l’anémie avant la grossesse ultérieure.

## Abstract (en)

**Introduction:**

Anemia is a public health problem, prevalent among children and women of childbearing age. Our study aims to determine the prevalence and factors associated with anemia in pregnant women at Douala General Hospital.

**Methods:**

We conducted a cross sectional study from July 2012 to July 2013. All consenting pregnant women attending antenatal consultation and having undergone complete blood count (CBC) were included in the study. Sociodemographic characteristics, individual’s obstetrical history and the results of the CBC were recorded on a pre tested data collection sheet. Anemia was defined according to the WHO criteria. After some descriptive statistics, we performed a bivariate analysis using the Chi-square test and Fisher exact probability test in order to determine the factors associated with anemia. P value <0.05 was considered significant.

**Results:**

A total of 415 pregnant women were enrolled in the study. Anemia prevalence was 39,8%. The average age was 29,89±4,835 years. The mean hemoglobin level was 10.93 ± 1.23. Normochromic normocytic anemia (53,3%) was prevalent. Anaemia was severe in 2,4% of cases. Anemia in pregnancy was significantly associated with a personal history of chronic diseases (P = 0.02) and of anemia in a previous pregnancy (P = 0.003). Anemia was more frequently observed during the 3rd trimester (P = 0.04) and breastfeeding played a protective role (P = 0.02).

**Conclusion:**

The prevalence of anemia during pregnancy remains high. A better management of chronic diseases in pregnant women and of postpartum follow-up is necessary to treat anemia before a subsequent pregnancy.

## Introduction

L’anémie définie par l’organisation mondiale de la santé(OMS) comme un taux d’hémoglobine inférieur à 11g/dl, est l’un des problèmes les plus courants en obstétrique. Elle est l’ultime expression d’une carence en fer qui en est la cause dans plus de 50% des cas; par ailleurs, il s’agit des causes infectieuses (paludisme et autres infections parasitaires), d’autres déficits nutritionnels, des anémies à hématies falciformes, aplasiques, inflammatoires et des anémies par perte de sang ; très souvent, l’origine est multifactorielle [1, 2]. Les besoins en fer durant la grossesse sont significativement augmentés surtout lors de la deuxième partie de la grossesse. Ceci est en rapportavec l’augmentation de la masse globulaire de la mère, les besoins du fœtus et du placenta,ainsi que les pertes sanguines au cours de l’accouchement. La réponse à ces besoins dépend de l’état des réserves avant la grossesse. L’OMS rapporte que 41,8% des femmes enceintes (pays développés et pays en voie de développement confondus) présentent une anémie [1]. Il s’agit d’un trouble de gravité variable auquel sont exposées 17 à 31% des femmes enceintes dans les pays développés et 52,8 à 61,3% en Afrique au Sud du Sahara [1]. Quelques études consultées rapportent des prévalences de 16,8%, 22,1%, 24,4%32,8%,41,6% et 100% respectivement en Iran, Ouganda, en Grande Bretagne,en Ethiopie, en Turkie et Inde [3–8]. Les facteurs de risques de l’anémie en grossesse varient considérablement d’un milieu à l’autre. Dans l’étude de Taner et al, Les facteurs retrouvés étaient la multiparité, le bas niveau d’éducation, un faible revenu mensuel, la consultation prénatale tardive et une courte durée de supplémentation en fer pendant la grossesse [7]. Dans l’étude Britanique de Barroso et al, il s’agissait du jeune âge, de l’origine ethnique autre que blancheet de la multiparité [5]. L’anémie est un facteur de risque significatif au regard de la morbidité maternelle et surtout fœtale (retard de croissance in utero, prématurité et mortalitépérinatale) [9]. Au regard de ces complications et compte tenu du fait qu’il s’agit d’une affection facilement corrigeable et évitable, nous nous sommes proposés de déterminer la prévalence et les facteurs associés de l´anémie chez les femmes enceintes à l’Hôpital Général de Douala. La détermination de ces facteurs va donner des renseignements sur les groupes à risquer ce qui permettra la mise en œuvre des interventions plus efficaces pour réduire l’anémie en grossesse.

## Méthodes

### Cadre d’étude

L’Hôpital Général de Douala (HGD) est un hôpital de référence, qui se classe dans la première catégorie, au sommet de la pyramide sanitaire. Le service de gynécologie-obstétrique site de notre étude est constitué de quatre sous-unités (bloc opératoire, salle d’accouchement, service de consultation externe, service d’hospitalisation) tenu par six médecins spécialistes, 24 infirmiers (5 sages-femmes, 7 infirmiers diplômés d´états,12 accoucheuses) et 4 auxiliaires de puéricultures. Les frais de consultation sont fixés à 7000 FCFA par consultation soit 70000 FCFA pour les 10 consultations et le cout du bilan prénatal minimal (biologie et échographies) effectué pendant le suivi d´une grossesse normale est d´environ 185750 FCFA. Les patientes recrutées à l ´Hôpital Général de Douala appartiennent à un quintile de bien-être économique riche et plus riche selon EnquêteDémographique de la Santé (EDS) 2011 [10].

**Type d’étude:** L’étude était transversale, descriptive et analytique sur une durée d´un an, du 1er juillet 2012 au 30 Juin 2013.

**Population d’étude:** Il s’agissait de toutes les femmes enceintes ayant consulté dans le service de gynécologie de l’HGD pendant la période d’étude.

**Critères d’inclusion et d’exclusion:** Nous avons inclus toutes les gestantes vue en consultation prénatale pendant la période d’étude, ayant une numération formule sanguine (NFS) réalisée à l’Hôpital Général de Douala. Etaient exclues, les gestantes ayant bénéficié d’une transfusion sanguine moins de trois mois avant leur consultation et celles qui avaient réalisé leur NFS en dehors de l’hôpital.

**Echantillonnage et taille de l’échantillon:** L’échantillonnage était consécutif. La taille d’échantillon a été calculée selon la formule de Lorenz en prenant la prévalence de l’anémie chez les femmes enceintes au Cameroun selon l´enquête démographique de santé en 2011 qui était de 49,9% [10]. Ce calcul a permis d’obtenir une taille d’échantillon de 384 patientes.

**Collecte des données:** Sur un total de 417 femmes enceintes remplissant les critères d’inclusion, 415 ont été retenues pour l’étude. Après obtention du consentement de la gestante, un questionnaire standardisé et pré-testé lui était administré. Les informations suivantes étaient recherchées: les paramètres socio démographiques (profession, niveaux scolaires, la situation matrimoniale); les antécédents de la patiente (Age gestationnel, la formule gravidique, métrorragie, ménorragie, l’hémorragie du post partum antérieure, l’anémie gravidique antérieure, l’hémorragie pendant la grossesse actuelle, l’allaitement maternel antérieur, la géophagie, l’espace inter génésique, l’antécédent de pathologies chroniques (pathologie ayant duré au moins trois mois ou pathologie incurable); les données biologiques: électrophorèse de l’hémoglobine; l’hémogramme ou NFS (taux d’hémoglobine, volume globulaire moyen (VGM) et la concentration corpusculaire moyenne en hémoglobine (CCMH)).

La NFS a été effectuée au laboratoire de l’HGD sur un automate d’hématologie de marque Ruby (abbott). L’anémie a été définie selon l´OMS c´est-à-dire un taux d’hémoglobine inferieur à 11g/dl chez la femme enceinte [1]. En fonction du degré de sévérité nous avons distingué: l’anémie légère: taux d’Hb entre 10,9 et 10g/dl; l’anémie modérée: taux d’Hb entre 7 et 9,9 g/dl; l’anémie sévère: taux d´Hb inferieur à 7g/dl. Le VGM normal a été défini par une valeur comprise entre 80 et 100 µ3. Sous le seuil de 80 µ3, nous avons parlé de microcytose et au-dessus de 100 µ3 de macrocytose. L’hypochromie a été définie par une CCMH≤32% et la normochromie par une CCMH > 32%.

**Analyse des données:** Les données ont été enregistrées et traitées à l’aide des logiciels EpiInfo 3.5.4 et Excel 2007. Les données ont ensuite été analysées à l’aide du logiciel SPSS. Les variables quantitatives ont été présentées en moyenne ± déviation standard (DS) et les variables qualitatives en effectifs et les pourcentages présentés entre parenthèses. En analyse bivariée, la comparaison entre les variables qualitatives a été effectuée à l’aide du test de Chi 2 et la probabilité exacte de Fisher a été déterminée dans le cas de variables dichotomiques et pour les échantillons de moins de 5. Les valeurs de la probabilité P <0.05 ont été considérées comme statistiquement significatives.

**Considérations éthiques:** Nous avions obtenu la clairance numéro 2013/05/305/CNERSH/SPdu comité national d’éthique de la recherche pour la santé humaine, donnant approbation de la réalisation de l’étude. Par ailleurs l’hôpital général de Douala avait donné une autorisation de recherche en son sein. Enfin chaque participante donnait son consentement éclaire afin d’être incluse. La confidentialité des informations a été assurée par la codification des fiches de collecte des données ; ces données ont été analysées anonymement.

## Résultats

### Caractéristiques sociodémographique population d´étude

Au total 415 patientes âgées de 18 à 44 ans ont été recrutées. L’âge moyen de ces patientes était de 29,89± 4,84 ans. La tranche d´âge la plus concernée était celle de 30 à 39 ans (n=208 cas soit 50,1%).Ces femmes étaient pour la plupart mariées (335 cas soit 80,7%) et avaient un niveau d’étude supérieur (279 cas soit 67,2%). Les femmes salariées étaient les plus représentées (240 cas soit 57,8%); (Tableau 1).

**Tableau 1:** Caractéristiques sociodémographiques des femmes étudiées

		Population totale	
		Effectif	%
Tranche d'âge	18-19	4	1,0
	20-29	193	46,5
	30-39	208	50,1
	40-44	10	2,4
Statut salarial	non salariée	175	42,2
	salariée	240	57,8
Niveau d’étude	Primaire	10	2,4
	Secondaire	126	30,4
	Universitaire	279	67,2
situation matrimoniale	Célibataire	80	19,3
	Mariée	335	80,7

### Distribution de l’anémie

Sur les 415 femmes, 165(39,8%) étaient anémiées. Parmi les femmes anémiées, l´anémie était légère chez 142 (86,1%) des cas et sévère dans 4(2.4%). L’anémie normochrome normocytaire était prédominante 88 (53,3%) cas suivi de l’anémie hypochrome microcytaire 33 (20,0%) cas (Tableau 2).

**Tableau 2:** Répartition en fonction du type d’anémie

Classification de l'anémie	Effectif	%
Anémie macrocytaire hypochrome	2	1,2
Anémie macrocytaire normo chrome	8	4,8
Anémie microcytaire hypochrome	33	20,0
Anémie microcytaire normochrome	16	9,7
Anémie normocytaire hypochrome	18	10,9
Anémie normocytaire normochrome	88	53,3
**Total**	**165**	**100,0**

### Facteurs associés à l’anémie

Il n’a pas été retrouvé d’association significative entre l’anémie et les facteurs sociodémographiques (Tableau 3). Par contre, les facteurssignificativement associés à la survenue de l´anémie en grossesse étaient l´antécédent d’absence d’allaitement maternel (p=0,02), la présence d’une pathologie chronique (p=0,02), l’antécédent d´anémie gravidique (p=0,01). L’anémie était plus fréquente au 3^ème^ trimestre de la grossesse (Tableau 4).

**Tableau 3:** Facteurs sociodémographiques associés à l'anémie

		Anémiées		Non Anémiées		P value	Odds ratio (IC)
		Effectif	%	Effectif	%		
Age	18-19	3	75	1	25	0,13	0,2 [0,02-1,99]
	20-29	73	37,8	120	62,2	1	
	30-39	84	40,4	124	59,6	0,60	0,90 [0,60-1,34]
	40-44	4	40	6	60	0,89	0,91 [0,25-3,34]
Statut salarial	Salarié	101	42,1	139	57,9		
	Non salarié	64	36,6	111	63,4	0,26	1,26 [0,84-1,88]
Niveau scolaire	Primaire et secondaire	62	45,6	74	54,4		
	Universitaire	102	36,6	177	63,4	0,08	0,69 [0,45-1,04]
Situation matrimoniale	Mariée	127	37,9	208	62,1		
	Célibataire	38	47,5	42	52,5	0,12	0,67 [0,41-1,10]

**Tableau 4:** Facteurs obstétricaux et médicaux associés à la grossesse

		Anémiées		non anémiées		P value	Odd Ratio (IC)
		Effectif	%	Effectif	%		
Parité	0-2	140	40,9	202	59,1	1	
	3-4	20	31,7	43	68,3	0,17	1,49 [0,84-2,84]
	5 et +	4	57,1	3	42,9	0,39	0,52 [0,11-2,36]
Espace inter génésique	≤2	60	40,8	87	59,2		
	>2	59	41,2	84	58,2	0,94	0,98 [0,61-1,57]
Allaitement maternel	Oui Non	99 13	40,6 68,4	145 6	59,4 31,6	**0,02**	**0,32 [0,12-0,86]**
Durée d’allaitement	0 – 6]	31	39,2	48	60,8	1	
(en mois)	] 6 – 12]	55	40,4	81	59,6	0,86	0,95 [0,54-1,68]
	> 12	11	40,7	16	59,3	0,89	0,94 [0,39-2,29]
Age gestationnel (en SA)	≤12	49	40,2	73	59,8	1	
	] 12-28]	86	35,7	155	64,3	0,40	1,21 [0,77-1,89]
	>28 SA	29	56,9	22	43,1	**0,04**	**1,96 [1,01**-**3,81]**
hémorragie pendant la grossesse	Oui	24	46,2	28	53,8		
	Non	138	38,4	221	61,6	0,26	1,4 [0,78-2,51]
ATCD d'anémie gravidique	Oui	23	62,2	14	37,8		
	Non	141	37,4	236	62,6	**0,003**	**2,75 [1,37**-**5,52]**
Gemellaire monofoetale		1 163	12,5 40,1	7 243	87,5 59,9	0,11	0,21 [0,03-1,75]
Pathologie chronique	Oui	18	60	12	40	**0,02**	
	Non	146	38,1	237	61,9		**2,43 [1,14**-**5,2]**
Pathologie	Autres+	3	60	2	40	1	
(Type)	HTA	4	50	4	50	0,72	1,5 [0,16-14,42]
	VIH	11	64,7	6	35,3	0,85	0,82 [0,11-6,34]

+ Il s’agissait du diabète et de l’hypothyroidie

## Discussion

Cette étude révèle que l’anémie est fréquente chez les femmes enceintes: 39,8% des patientes recrutées avaient une anémie. Cette fréquence est basse par rapport à celle retrouvée sur le plan national (49,9%) [10]. La différence peut venir du fait qu’il s’agissait d’une étude unicentrique. Par ailleurs, il s’agit globalement d’une population assez instruite (pas de patiente analphabète et2,4% de niveau primaire), ce qui pourrait être en faveur des meilleurs connaissances sur les carences alimentaires. La carence en fer étant la première cause de l’anémie en grossesse. Cette prévalence de 39,8% est superposable à celle deDesalegn et alen Ethiopie et Taner et al en Turkie qui ont retrouvé respectivement 41,9% et 41,6% [7, 11]. Des prévalences plus basses ont été rapportées en Iran (16,8%), en Ouganda (22,1%) et en Grande Bretagne (24,4%) [3–5]. Selon les statistiques de l’OMS, la prévalence de l’anémie est liée au développement et reste plus basse dans les pays développés. On peut penser que l’Ouganda fait l’exception, mais l’étude dont il est question ici a été réalisée dans 2formations sanitaires, une dans le Nord (très pauvre) où la prévalence était de 32,9% et l’autre dans l’Ouest plus développé avec une prévalence de 12,1% [4]. Une autre étude menée au centre hospitalier universitaire de Kano au Nigéria a rapporté une prévalence basse (17%) probablement parce qu’il s’agissait d’une étude monocentrique (sur 300 femmes)qui ne reflète pas la réalité nationale [12]. Ce d’autant que dans le même pays, d’autres auteurs ont rapporté une prévalence de 51,8% [13]. D’une manière générale, la prévalence del’anémie en grossesse est plus élevée dans les pays en développement que celle rapportée par notre étude, ainsi nous avons retrouvé dans la littérature des prévalences allant de 50% à 100% [8, 14, 15].

L’anémie normocytaire normo chrome était la plus fréquente (53,3%) et la carence en fer pouvait être évoquée dans 20% des cas au travers de l’hypochromie et de la microcytose. 20,6% des patientes avaient une anémie avec soit une hypocromie isolée ou une microcytose isolée, ce qui peutévoquer aussi une carence en fer. Quoi qu’il en soit, l’idéal aurait été de doser le fer sérique et la ferritine pour mieux identifier l’anémie ferriprive. L’anémie normo chrome normocytaire pourrait être en rapport avec l´hémodilution habituellement observée pendant la grossesse. Nwizu au Nigéria avait une fréquence de l’anémie hypochrome microcytaire de 11,8 % des cas [12]. L’anémie était en générale légère ou modérée avec les fréquences respectives de 86,1 % et 11,5 %. D’autres auteurs ont également noté une forte proportion d’anémie légère et modérée [4, 8, 13, 14].

### Facteurs sociodémographiques associés à l’anémie

**Age:** Certaines études mettent en évidence, une augmentation de la prévalence de l’anémie en grossesse chez les adolescentes [16, 17]. Notons que dans ces études, l´influence de l´âge n´a souvent pas été séparée de l´effet de la parité et de l´état nutritionnel pré-grossesse, facteurs identifiés comme a risque d´anémie chez les adolescentes enceintes. Les adolescentes étaient très faiblement représentées dans notre étude (4 cas).Par ailleurs, il n’y avait pasd’association significative entre la survenue de l’anémie et les différentes tranches d’âge, tout comme dans le travail de Taner et al et Nwizu et al [7, 12].

**Niveau économique:** Les femmes salariées étaient susceptible d´être plus anémiées (42,1%) que chez les femmes non salariées (36,6%) cependant la différence n’était pas significative sur le plan statistique.Le bas niveau socio-économique est associé à l’anémie en grossesse [6, 7, 13, 14], mais l’absence de salaire dans notre étude ne signifie pas bas forcément bas niveau socio-économique, car très souvent, les conjoints de ces femmes non salariées travaillent et arrivent à couvrir les besoins de la famille.

**Niveaux d´instruction:** Les femmes ayant le niveau secondaire étaient plus anémiées (47,6%) que les femmes ayant un niveau universitaire (36,7%), différence proche du seuil de significativité (p 0,08). Bekele et al en Ethiopie n’ont pas trouvé d’association en le niveau d’éducation et l’anémie chez la femme enceinte non plus. Ces trouvailles sont contraires à celles de la plus des auteurs pour qui le bas niveau d’éducation est un facteur significativement associé à l’anémie en grossesse [7, 12, 14, 18]. Ceci s’explique par le fait que la femme illettrée ne fera pas assez attention à son régime alimentaire. Par ailleurs, très souvent elle appartient à la basse classe sociale avec faible revenu. Ceci entraine un risque de malnutrition (faible pouvoir d’achat) et d’accès limité aux soins de santé dans un environnement où il n’existe pas d’assurance maladie pour tous ou de couverture sanitaire sociale.

**Situation matrimoniale:** La fréquence de l’anémie n’était pas statistiquement plus élevée chez les femmes célibataires (46,8%) que chez lesmariées (37,9%). Selon Nwizu [9] et collau Nigeria, les femmes célibataires étaient significativement plus susceptibles d´être anémiées que les mariées(OR : 2,02). Les femmes seraient sont soutenues moralement et financièrement par leur conjoint, ce qui peut expliquer cette différence. Cependant, dans d’autres travaux, l’association n’est pas retrouvées [4, 6] d’où la nécessité de faire des études randomisées à plus large échelle.

### Facteursobstétricaux et médicaux associés à l´anémie

**Parité:** La prévalence de l´anémie est plus élevée chez les grandes multipares (57,1%) comparées aux paucipares (40,9%),il en est de même dans certaines études [6, 7, 12] qui trouvent que le risque augmente avec la parité. Ceci peut s’expliquer par l’absence de compensation des pertes et la déplétion des réserves enregistrées lors des grossesses et allaitements précédents. Ce résultat est contradictoire à celui d’autres études Africaines qui trouvent une prévalence plus élevée chez la primipare et l’attribue à leur susceptibilité accrue au paludisme [19, 20].

**Âge gestationnel:** Le risque accru d’anémie chez les femmes au troisième trimestre de grossesse observé (56,9%) est similaire aux résultats de plusieurs autres études [7, 12, 14, 19] qui trouvent que la proportion de l´anémie augmente au deuxième et troisième trimestre. Cela pourrait s´expliquer par l’hémodilution accrue dès le deuxième trimestre de grossesse.

**Pathologie chronique:** La prévalence de l´anémie chez les femmes enceintes ayant une pathologie chronique est de 60% (p=0,02). Ainsi nous pourrons penser comme dans certaines études [14, 21] que l´état d´anémie est aggravé par la présence de maladies chroniques. Dans les régions d´endémie palustre, le paludisme peut provoquer une anémie hémolytique aigue au cours de la grossesse. Par ailleurs, la prévalence de l´anémie chez les femmes immunodéprimées au VIH était de 64,7% dans notre étude. Le VIH provoque plusieurs manifestations hématologiques notamment la thrombocytopénie immunitaire, la coagulation et l´anémie. L´anémie ici est une conséquence de l´inflammation, la suppression de la moelle osseuse, une hémolyse ou d´une thérapie antirétrovirale [22].

**Hémorragie pendant la grossesse:** le risque d´anémie induite est variable selon l’importance de la perte sanguine. Dans notre étude, comme dans celle de Taner et al [7], l´anémie en grossesse n’était pas significativement liée àl’hémorragie anténatale.

**Antécédent d´anémiegravidique:** La prévalence de l´anémie chez les femmes ayant un antécédent d´anémie gravidique était de 62,2% (p=0,01).Ceci s’expliquerait par le fait que ces femmes n’ont pas eu le temps de compenser leur déficit avant la nouvelle grossesse soit en raison d’une alimentation pauvre en fer et folate, ou des maladies intercurrentes telles que le paludisme ou enfin d’un espace inter génésique court (< 2ans). En effet certains auteurs ont retrouvé une association entre les grossesses rapprochées (espace intergénésique inférieur à 2 ans) et l’anémie en grossesse [6, 12].

**Allaitement maternel:** L’allaitement maternel apparait ici comme un facteur protecteur de l’anémie, beaucoup d’auteurs n’ont pas analysés ce facteur. Les explications à ces trouvailles pourraient être premièrement, le nombre limité de femmes qui n’a pas allaité, deuxièmement dans les coutumes au Cameroun, la femme qui allaite doit bien s’alimenter et boire beaucoup de lait, ce qui pourrait en fait accélérerla récupération des pertes observées pendant la grossesse et l’accouchement (alimentation plus diversifiée et plus équilibrée).

**Grossesse multiple:** La prévalence de l´anémie chez les femmes ayant une grossesse gémellaire était de 12,5%.Par contre, une étude menée par Gulrukh [23] a montré que l´anémie représentait 74,6% des complications observées chez les femmes ayant une grossesse gémellaire. Dans notre contexte, cette différence est due au faible effectif des femmes ayant une grossesse gémellaire.

## Conclusion

L’anémie pendant la grossesse reste encore un problème de santé publique. La présence de pathologies chroniques et l'antécédent d'anémie gravidique sont significativement associés à l'anémie pendant la grossesse dans cette population. Cette anémie est plus fréquente au 3^ème^ trimestre. L’allaitement maternel est un facteur protecteur. Nos données renforcent les besoins d'une stratégie d'intervention globale visant à bien prendre en charge les pathologies pouvant se compliquer d’une anémie et à bien suivre les parturientes dans le post partum afin de corriger leur déficit avant la prochaine grossesse. **Limite:** Cette étude a été réalisée dans un hôpital de référence où le pouvoir d’achat des patientes ne reflète pas celui de la population générale. On ne s’aurait donc généraliser les résultats.Par ailleurs, le bilan complémentaire de l’anémie aurait pu nous donner de meilleures précisions étiologiques. Néanmoins, ces résultats sont le reflet de l’anémie chez les gestantes de classe économique élevée ou moyenne à Douala.

### Etat des connaissances actuelles sur le sujet

- L’anémie est un problème de santé publique, prédominant chez les enfants et les femmes en âge de procréer;
- L’OMS rapporte un taux d’anémie en grossesse plus élevé dans les pays en voie de développement (52,8 à 61,3%) par rapport aux pays développés (17 à 31%);
- Les facteurs de risques de l’anémie en grossesse varient considérablement d’un milieu à l’autre. Ils peuvent être : la multiparité, le bas niveau d’éducation, un faible revenu mensuel, la consultation prénatale tardive et une courte durée de supplémentation en fer pendant la grossesse.

### Contribution de notre étude à la connaissance

- La prévalence de l’anémie en grossesse dans un hôpital tertiaire à Douala (39,8% dans cette étude);
- Les facteurs associés à l’anémie en grossesse à Douala: La présence de pathologies chroniques et l'antécédent d'anémie gravidique étaient significativement associés à cette anémie en grossesse;
- L’allaitement maternel comme facteur protecteur de l’anémie en grossesse.
